# Antibodies against Alpha-Synuclein Reduce Oligomerization in Living Cells

**DOI:** 10.1371/journal.pone.0027230

**Published:** 2011-10-31

**Authors:** Thomas Näsström, Susana Gonçalves, Charlotte Sahlin, Eva Nordström, Valentina Screpanti Sundquist, Lars Lannfelt, Joakim Bergström, Tiago F. Outeiro, Martin Ingelsson

**Affiliations:** 1 Rudbeck Laboratory, Department of Public Health/Geriatrics, Uppsala University, Uppsala, Sweden; 2 Cell and Molecular Neuroscience Unit, Instituto de Medicina Molecular, Lisboa, Portugal; 3 Instituto de Fisiologia, Faculdade de Medicina da Universidade de Lisboa, Lisboa, Portugal; 4 BioArctic Neuroscience AB, Stockholm, Sweden; 5 Department of NeuroDegeneration and Restaurative Research, Universitätsmedizin Göttingen, Göttingen, Germany; University of Kent, United Kingdom

## Abstract

Recent research implicates soluble aggregated forms of α-synuclein as neurotoxic species with a central role in the pathogenesis of Parkinson's disease and related disorders. The pathway by which α-synuclein aggregates is believed to follow a step-wise pattern, in which dimers and smaller oligomers are initially formed. Here, we used H4 neuroglioma cells expressing α-synuclein fused to hemi:GFP constructs to study the effects of α-synuclein monoclonal antibodies on the early stages of aggregation, as quantified by Bimolecular Fluorescence Complementation assay. Widefield and confocal microscopy revealed that cells treated for 48 h with monoclonal antibodies internalized antibodies to various degrees. C-terminal and oligomer-selective α-synuclein antibodies reduced the extent of α-synuclein dimerization/oligomerization, as indicated by decreased GFP fluorescence signal. Furthermore, ELISA measurements on lysates and conditioned media from antibody treated cells displayed lower α-synuclein levels compared to untreated cells, suggesting increased protein turnover. Taken together, our results propose that extracellular administration of monoclonal antibodies can modify or inhibit early steps in the aggregation process of α-synuclein, thus providing further support for passive immunization against diseases with α-synuclein pathology.

## Introduction

Parkinson's disease, dementia with Lewy bodies and multiple system atrophy are neurodegenerative disorders characterized by the loss of neurons in the brain along with the presence of large intracellular protein inclusions known as Lewy bodies [1], [2]. The major protein component of Lewy bodies is α-synuclein, a 140 amino acid long protein with a partially unfolded structure [3]. Although α-synuclein has a largely unknown function, recent findings suggest it to be involved in neurotransmitter regulation. For example, α-synuclein may regulate the reuptake of dopamine into striatum of transgenic mice [4] or be more generally involved in synaptic release by promoting SNARE complex assembly [5].

The aggregation cascade of α-synuclein is believed to begin with the formation of dimers and smaller oligomers before the appearance of larger oligomers or protofibrils [6]. Such soluble pre-aggregated species have been demonstrated to have toxic properties and may thus play a central role in the pathogenesis [7], [8], [9], [10], [11]. In addition, the disease associated mutations in the gene encoding for α-synuclein have been found to increase the formation of oligomers/protofibrils, further supporting the pathogenic significance of such species [12], [13], [14].

Alpha-synuclein aggregation has been widely studied in cell culture models. By overexpressing α-synuclein, intracellular inclusions can be induced in a wide range of cell types via various aggregation-promoting conditions [15], [16]. Early stages of protein aggregation can be assessed with protein-fragment complementation techniques [17]. One such method, the bimolecular fluorescence complementation (BiFC) assay, has previously been adopted for the study of α-synuclein aggregation [7]. By fusing DNA encoding either the C-terminal or N-terminal halves of GFP to the entire α-synuclein sequence, two forms of α-synuclein hemi:GFP constructs are generated. Upon double transfection of cells with these constructs, fluorescence arises only when the fragments are brought together, i.e. after dimerization/oligomerization of α-synuclein.

In the last decade, immunotherapy has emerged as a promising tool to target and clear protein pathology in neurodegenerative diseases. With active immunization of transgenic amyloid-beta precursor protein (AβPP) mice, using fibrils of the amyloid beta peptide (Aβ), a distinct reduction of Aβ pathology could be seen [18]. In addition, Aβ immunization has been found to alleviate memory impairment in transgenic animal models [19]. Instead of vaccination in Alzheimer's disease, focus has now been set on passive treatment with antibodies against Aβ. Such an approach has proven to be equally efficient in both cell and animal models and is likely to be a safer therapeutic option, as T-cell mediated side effects can be avoided [20], [21].

Immunotherapy has now also begun to be evaluated as an approach to treat α-synuclein pathology. In one study, active immunization with α-synuclein on transgenic mice showed that the pathology was less pronounced in vaccinated mice as compared to placebo [22]. As for passive immunotherapy against α-synuclein pathology, a recent study described reduced behavioral deficit as well as decreased accumulation of α-synuclein aggregates in an α-synuclein transgenic mouse model [23].

Here, we explored the use of monoclonal α-synuclein antibodies to target dimerization/oligomerization on a cell culture model, using BiFC.

## Materials and Methods

### Alpha-synuclein constructs

The G-N-155-α-syn and α-syn-G-156-C constructs used for the BiFC assay were generated as described earlier [7]. For all transfection experiments, an empty pcDNA3.1 expression vector (Invitrogen, Carlsbad, CA) was used as control.

### Cell culture

Human H4 neuroglioma cells were a kind gift of Dr. Bradley T. Hyman (Massachusetts General Hospital, Charlestown, MA). Cells were cultured at 37°C and 5% CO_2_ in OPTI-MEM (Invitrogen) and supplemented with 10% fetal bovine serum (FBS) (Invitrogen) and 4 mM Glutamine (Invitrogen).

### Antibodies

The following α-synuclein monoclonal antibodies (mAb) were used for cell culture treatment: mAb211 (Santa Cruz Biotechnology, Santa Cruz, CA), mAb5C2 (Santa Cruz Biotechnology) and the oligomer-selective antibody mAb49/G (BioArctic Neuroscience, Stockholm, Sweden). The monoclonal GAPDH antibody 9484 (Abcam, Cambridge, UK) was used as a negative treatment control. All antibodies used for cellular treatment were diluted in TBS to reach a final concentration of 1 µg/ml in the extracellular media. For the sandwich ELISA, the Syn-1 (BD Biosciences, Franklin Lakes, NJ) and FL-140 (Santa Cruz Biotechnology) α-synuclein antibodies were used for capture and detection, respectively. For immunocytochemistry experiments, anti-mouse Cy3 or Alexa594 conjugated secondary antibodies (Invitrogen) were used.

### Generation of the oligomer-selective α-synuclein antibody mAb49/G

Balb/c mice (The Jackson Laboratory, Bar Harbor, Maine) were immunized with 4-hydroxy-2-nonenal (HNE) stabilized α-synuclein oligomers [11] diluted 1∶1 (v:v) with Freund's adjuvant. After repeated boosts, immunized mice with high serum titers were sacrificed for isolation of spleen cells. Next, the spleen cells were fused with SP2/0 myeloma cells. Hybridomas were screened for anti-α-synuclein reactivity with ELISA and positive clones underwent at least two rounds of limiting dilution assay to ensure monoclonality. The IsoStrip kit (Roche Diagnostics, Basel, Switzerland) was used to determine isotype and subclass of the antibody. The mAb49/G IgG_1_ antibody was then purified from the conditioned media with affinity chromatography using Protein G-Sepharose (GE Healthcare, Uppsala, Sweden). All experiments involving animals were approved by the local ethical committee (Stockholms Norra djurförsöksetiska nämnd, decision numbers N417/08; 2009-01-15).

### Inhibition ELISA

An inhibition ELISA assay was performed as described previously using α-synuclein monomer coated plates. As competitors, α-synuclein monomers and HNE stabilized α-synuclein oligomers were used [24].

### Transfection and addition of monoclonal antibodies

Prior to the day of transfection, cells were seeded onto 35 mm poly-D-lysine coated culture plates (MatTek Cultureware, Ashland, MA) at a density of 1,5×10^5^ cells/plate. Transfection of H4 cells were carried out with a 1∶5 ratio (µg DNA:µl FuGENE), using the FuGENE 6 Transfection reagent (Roche Diagnostics). In brief, the culture medium was replaced with medium containing 1% FBS, transfected and left to incubate at 37°C for 24 h. For bioimaging, to ensure optimal reconstitution of the two GFP fragments, cells were incubated over night at 30°C [7], [25]. Moreover, cells were treated or untreated at time zero of transfection with either of the mAb211, mAb5C2 or mAb49/G α-synuclein antibodies or with the mAb9484 GAPDH antibody for 48 h at a final antibody concentration of 1 µg/ml.

### Immunocytochemistry

Cells were washed with PBS and subsequently fixed for 10 min in 4% paraformaldehyde (PFA). After permeabilization for 20 min in 0.1% Triton X-100 at room temperature, the cells were blocked with 1.5% normal goat serum (NGS) (Invitrogen) for 2 h. After washing with PBS, cells were incubated with a Cy3 conjugated secondary antibody (Invitrogen) (1∶5000 in 1.5% NGS) for 1 h. Finally, cells were stained with DAPI (Invitrogen) (1∶20000 in 1.5% NGS) for 10 min.

Control cells were stained using the mouse monoclonal α-synuclein antibodies mAb49/G (BioArctic Neuroscience), mAb211 (Santa Cruz Biotechnology) and mAb5C2 (Santa Cruz Biotechnology) (1∶500 in 1.5% NGS) for 2 h. Next, cells were probed with the Alexa-Fluor 594 conjugated secondary antibody (Invitrogen) (1∶5000 in 1.5% NGS) for 1 h. Finally, cells were stained with DAPI (Invitrogen) (1∶20000 in 1.5% NGS) for 10 min. All incubations were performed at room temperature.

To control for unspecific binding of the secondary antibodies, cells were treated only with a mouse secondary antibody and compared to buffer treated controls. To control for passive uptake of the α-synuclein antibodies as an effect of DNA-transfection, additional experiments in which the antibodies were added with and without simultaneous administration of FuGENE 6 were carried out.

Cells were analyzed with confocal microscopy using a LSM 510 META instrument (Carl Zeiss Microimaging) where single plane and z-slice images of antibody internalization were obtained.

### Fixing cells and fluorescence microscopy

At the end of the treatment, cells were washed with PBS and subsequently fixed for 10 min in 4% paraformaldehyde (PFA). To study GFP fluorescence the cells were analyzed using an Axiovert 200 M widefield fluorescence microscope (Carl Zeiss Microimaging GmbH, Jena, Germany). The cells were observed using an Epi-fluorescence illuminator equipped with a FITC filter. Eight random sites in the well for each condition were observed using a 20X objective.

### Quantification of fluorescence intensity and image processing

For quantification of pixel intensities, the ImageJ (NIH, Bethesda, MD) software was used. The GFP fluorescence was converted to average pixel intensities for each condition. The intensities for each captured frame are presented as fold increase in fluorescence over vector transfected controls. A standard threshold for the highest exposure of positive pixels was set for the green channel by using α-synuclein–BiFC expressing cells without antibody treatment (-mAb). To test for statistically significant differences, groups were subjected to one-way ANOVA. Probability values <0.05 were considered significant using a two-tailed confidence interval.

### Preparation of conditioned media and cell lysates

Forty-eight hours after transfection and antibody treatment, the conditioned media was recovered and centrifuged at 2150 x g at 4°C for 10 min. To concentrate the samples, the conditioned media was freeze-dried and re-dissolved in CellyticM (Sigma-Aldrich, St. Louis, MO) lysis buffer supplemented with protease inhibitor cocktail (Roche Diagnostics). The cells were washed with PBS, lysed with CellyticM (Sigma-Aldrich) and supplemented with a protease inhibitor cocktail (Roche Diagnostics). The lysate was collected and centrifuged at 4°C for 10 min and 20800 x g. Protein concentrations in conditioned media and lysates were determined with the BCA Protein Assay Reagent (Thermo Fisher Scientific, Rockford, IL).

### Sandwich ELISA

A 96-well high binding plate polystyrene microtiter plate (Corning) was coated with 200 ng/well of Syn-1 (BD Biosciences) in PBS and incubated at 4°C over night. The solution was removed from each well and the cells were blocked with 1% BSA/0.15% Kathon for 1 h at room temperature. The samples, including a standard series of recombinant α-synuclein diluted in 1% BSA, 0.05% Tween and 0.15% Kathon, were added to the wells and incubated with shaking at room temperature for 2 h. After washing, the FL-140 polyclonal α-synuclein antibody (Santa Cruz Biotechnology) was diluted to 1 µg/ml and added to the wells, followed by shaking at room temperature for 1 h. Next, the wells were washed and an anti-rabbit horse radish peroxidase (HRP) conjugated detection antibody (Pierce, Rockford, IL, USA) was added at a final concentration of 0.4 µg/ml. After a further incubation for 1 h, the wells were washed and the K-blue aqueous substrate (TMB) was used as substrate for HRP. Before measurement, the reaction was stopped using 1 M H_2_SO_4_. The plate was measured using a SpectraMAX 190 (Molecular Devices, Palo Alto, CA) spectrophotometer at 450 nm. The data for each sample was calculated as the means of three separate wells.

## Results

### Characterization of mAb49/G

**Figure 1:**
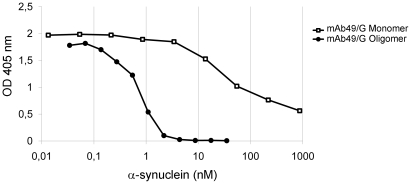
Characterization of mAb49/G by inhibition ELISA. Binding of mAb49/G to α-synuclein monomers (□) or α-synuclein oligomers (•) was analyzed on HNE stabilized α-synuclein oligomer coated plates. On the x-axis, the molar concentration of α-synuclein is displayed. The IC50 values are calculated as the concentration of either α-synuclein monomers or α-synuclein oligomers needed to quench half of the signal in the ELISA. Note that, due to uncertainties concerning the size of the α-synuclein oligomers used in this assay, the concentration in pM for both species is based on the molecular weight of one α-synuclein monomer. These data are representative of at least three independent experiments.

Immunization of mice with HNE stabilized α-synuclein oligomers resulted in several monoclonal antibodies, among which mAb49/G was chosen for this study. By inhibition ELISA, the binding of mAb49/G to an HNE stabilized α-synuclein oligomer coated plate was inhibited by addition of serially diluted α-synuclein species. When adding α-synuclein oligomers, the IC50 levels were in the low nanomolar range (0.7 nM) whereas the addition of at least 80 nM α-synuclein monomers were needed to quench the same signal, indicating a strong selectivity of mAb49/G for oligomeric α-synuclein (Fig. 1).

### Cellular internalization of the α-synuclein antibodies

**Figure 2:**
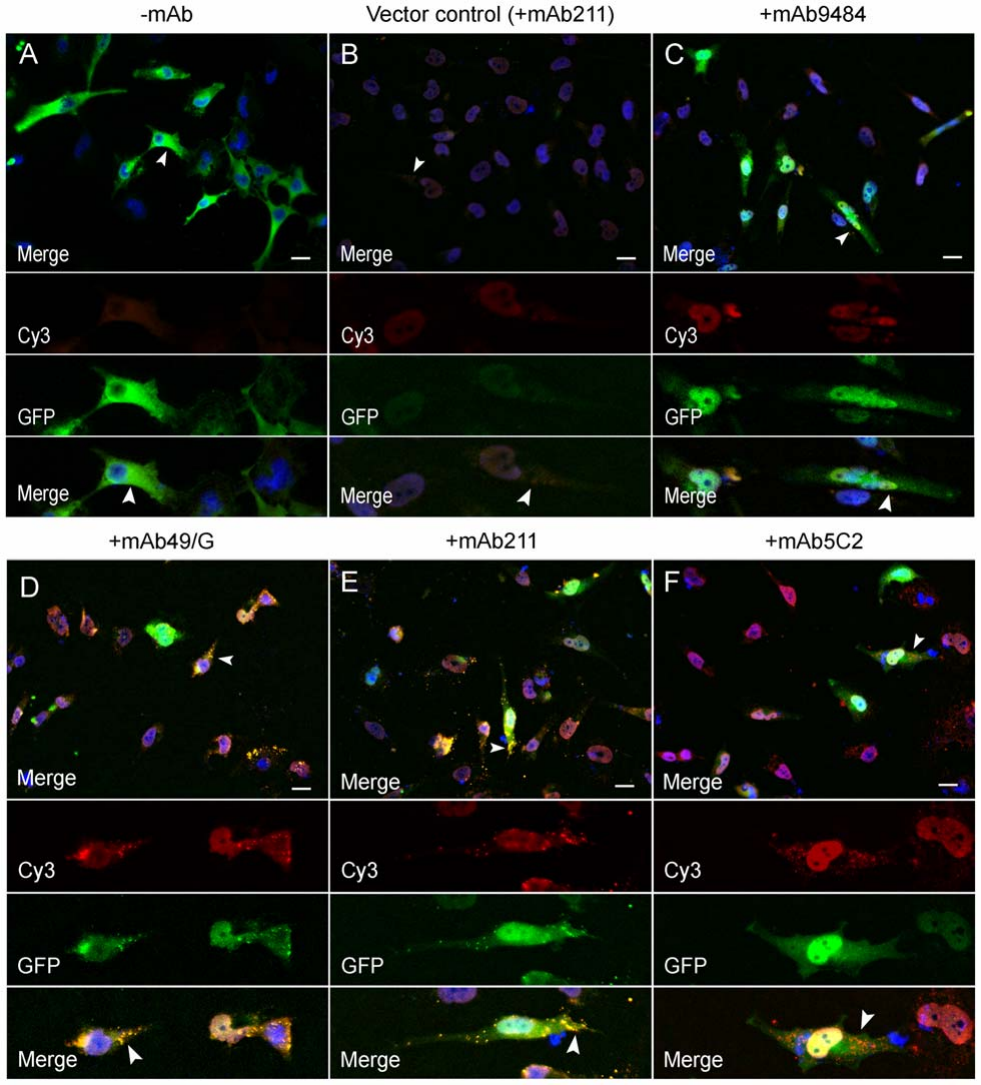
Immunocytochemistry with anti-mouse secondary antibodies (Cy3) displays internalization of the α-synuclein monoclonal antibodies (+mAb). Forty-eight hours after transfection with the two α-synuclein hemi:GFP constructs, cells displayed GFP fluorescence in the whole cell soma (green) (Fig. A). Cells transfected with the constructs and treated with the α-synuclein antibodies mAb49/G and mAb211 displayed less diffuse GFP fluorescence but more localized GFP-punctae (Fig. D, E, arrows). These signals occasionally co-occurred with signals from an anti-mouse secondary antibody, indicating internalization (yellow) of the treatment antibodies (Fig. D, E, arrows). Cells treated with the mAb5C2 antibody only displayed diffuse GFP-fluorescence in the whole cell soma (Fig. F). After staining with an anti-mouse secondary antibody red punctae could be detected in the cells indicating no co-occurrence with GFP (Fig. F, arrows). The blue signal represents DAPI nuclear staining (40x magnification. Scale bar 20 µm).

Antibody uptake was studied with immunocytochemistry followed by widefield and confocal microscopy. After 48 h of transfection with the two α-synuclein hemi:GFP constructs, GFP fluorescence was detected with widefield microscopy indicating dimerization/oligomerization of α-synuclein (Fig. 2A). In parallel experiments, cells transfected with the two α-synuclein hemi:GFP constructs and immediately treated with the mAb49/G, mAb211 and mAb5C2 antibodies for 48 h, displayed occasional small punctae of α-synuclein in the cell soma after immunostaining with secondary antibodies (Fig. 2D, E and F, arrows). Interestingly, these punctae partly co-occurred with GFP-positive signals, indicating internalization of the extracellularly added α-synuclein antibodies mAb49/G and mAb211 (Fig. 2D, E, arrows). However, by examining inclusion staining for the 5C2 antibody, cells exhibited red signals with no co-occurring GFP-fluorescence indicating binding to monomeric forms of the α-synuclein hemi:GFP for this particular antibody (Fig. 2F, arrows).

**Figure 3:**
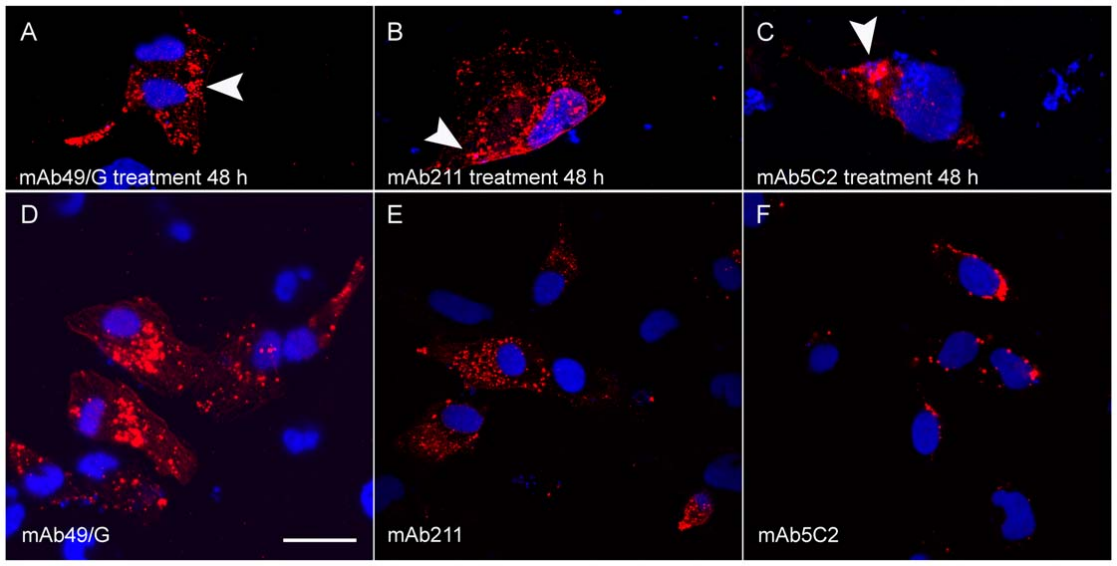
Confocal microscopy showing internalization of the α-synuclein antibodies. Forty-eight hours after addition of the mAb49/G, mAb211 and mAb5C2 α-synuclein antibodies, red punctate structures were detected within the cells after staining with anti-mouse secondary antibodies (Fig A, B and C, arrows). For comparison, immunocytochemistry was carried out using mAb49/G, mAb211 and mAb5C2 as primary antibodies (Fig. D,E and F). The blue signal represents DAPI nuclear staining (63x magnification. Scale bar 20 µm).

To confirm antibody uptake, cells were also analyzed by confocal microscopy. All antibodies were then found to get internalized by obtaining z-slice images, but the intracellular presence of mAb49/G and mAb211 was especially pronounced (Fig. 3A, B and C, arrows). Moreover, the antibodies labeled several small inclusions throughout the cell soma but were not found to stain the nucleus (Fig. 3A–C). For comparison, cells were subjected to ordinary immunocytochemistry using the mAb49/G, mAb211 and 5C2 as primary antibodies (Fig. 3D–F). Indeed, a similar pattern of immunofluorescence staining was detected in those cells confirming antibody localization to small inclusions of α-synuclein in the cell soma (Fig. 3A–C).

To control for passive uptake of the antibodies as a result of DNA transfection, we performed experiments in which the antibodies were incubated with or without the presence of FuGENE 6. However, we could not see any difference in antibody uptake between these two experimental conditions, thus indicating that the antibody uptake was not dependent on the presence of cell permeabilization reagents (data not shown).

### Reduced oligomerization after treatment with C-terminal specific and oligomer selective α-synuclein antibodies

**Figure 4:**
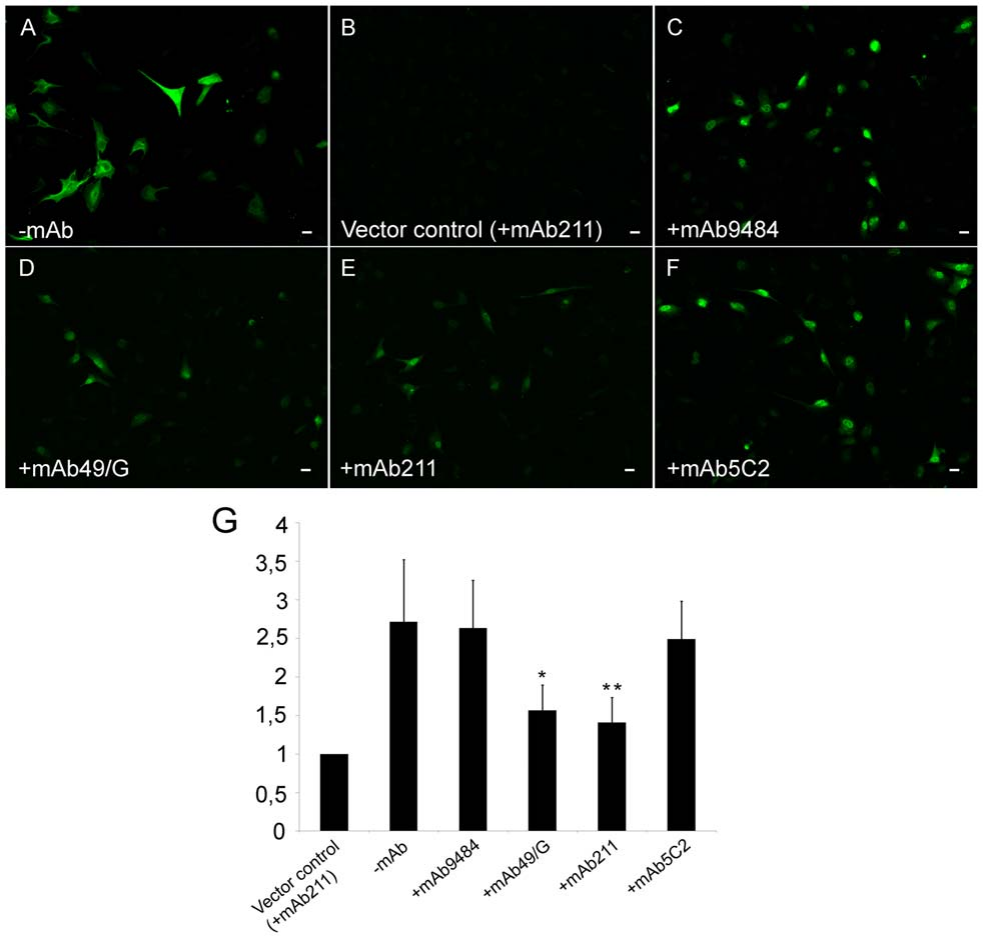
Alpha-synuclein dimerization/oligomerization, as shown by GFP fluorescence reconstitution. Forty eight hours after double transfection with the two α-synuclein hemi:GFP constructs, the H4 cells exhibited robust GFP fluorescence (2.7-fold over expression to vector controls) throughout the cell soma and nucleus (A and G). When cells were treated (+mAb) with the α-synuclein C-terminal specific antibodies mAb49/G and mAb211 the GFP fluorescence was significantly (*p<0.05, **p<0.01) reduced (1.4- and 1.5-fold over expression to vector controls respectively) indicating less dimerization/oligomerization (Fig. D, E and G). The mAb5C2 antibody targeting the non-Aβ component (NAC) region of α-synuclein did not show any reduction (2.5-fold over expression to vector controls) of GFP fluorescence, indicating no effect on the formation of dimers/oligomers (Fig. F and G). With the monoclonal antibody mAb9484 against GAPDH, no apparent effect (2.6-fold over expression to vector controls) on dimerization/oligomerization could be seen (Fig. C and G). 20X magnification. Scale bar 20 µm.

H4 neuroglioma cells were transfected with the two α-synuclein hemi:GFP constructs. Forty eight hours after transfection, GFP fluorescence could be detected in the cell soma and the nucleus in approximately 50% of the cells, indicating α-synuclein dimerization/oligomerization (Fig. 4A). The fluorescence in α-synuclein hemi:GFP transfected cells corresponded to a robust increase in fluorescence (2.7-fold over expression to vector controls).

In parallel experiments, α-synuclein antibodies were added to the cell media immediately after transfection. Addition of the α-synuclein C-terminal antibodies mAb49/G and mAb211 reduced the GFP fluorescence significantly (1.4- and 1.5-fold over expression to vector controls, respectively, p<0.05, p<0.01) (Fig. 4D, E and G). With the α-synuclein mid-region antibody mAb5C2, raised against the non-Aβ component (NAC) region, there was no reduction (2.5-fold over expression to vector controls) of GFP fluorescence (Fig. 4C and G).

Thus, treatment with C-terminal α-synuclein antibodies resulted in decreased formation of α-synuclein dimers/oligomers (Fig. 4D and E), whereas treatment with the mid-region antibody mAb5C2 did not seem to affect the extent of α-synuclein oligomerization. To ensure that the effects were specific to the α-synuclein antibodies, the monoclonal GAPDH antibody mAb9484 was added in parallel (Fig. 4C). No apparent decrease (2.6-fold over expression to vector controls) of GFP fluorescence was seen with this antibody, thus indicating that α-synuclein oligomerization was not affected by an irrelevant monoclonal antibody (Fig. 4C and G).

### Decreased intra and extracellular levels of α-synuclein from antibody treated cells

**Figure 5:**
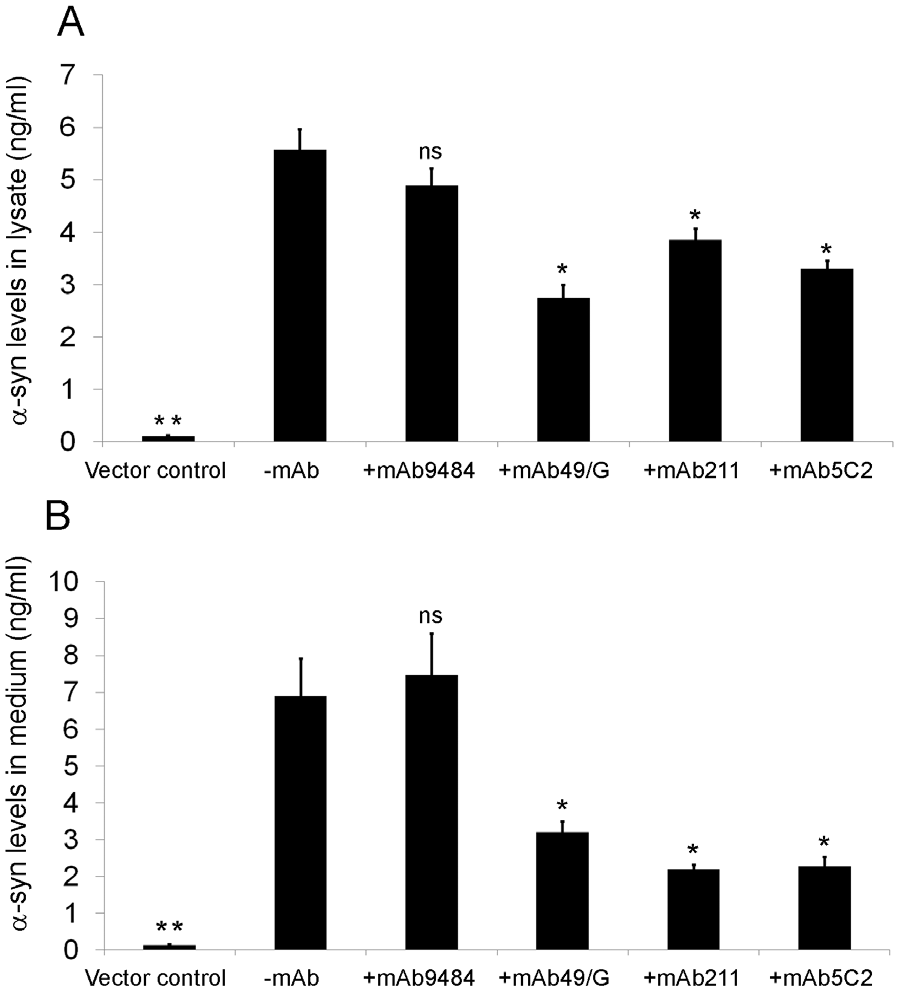
ELISA measurement of α-synuclein levels in lysates and media from BiFC expressing cells. In lysate from BiFC expressing cells (-mAb), the levels were 5.6 ng/ml (Fig. A), with mAb49/G treatment (+mAb) 2.8 ng/ml (*p<0.04), mAb211 treatment 3.9 ng/ml (*p<0.05) and with mAb5C2 treatment 3.3 ng/ml (*p<0.05) showing a reduction in protein content (Fig. A). In conditioned media from untreated cells (-mAb) the α-synuclein levels were 6.9 ng/ml (Fig. B). In media from antibody treated cells (+mAb), the levels were 3.2 ng/ml for mAb49/G (*p<0.05), 2.2 ng/ml for mAb211 (*p<0.05) and 2.3 ng/ml (*p<0.05) with mAb5C2 (Fig. B).

A sandwich ELISA was used to measure α-synuclein levels in lysate and conditioned media from the same cells that were analyzed for BiFC. In lysate from BiFC expressing cells (with no mAb added), α-synuclein levels were calculated to 5.6 ng/ml (Fig. 5A). In comparison, levels of α-synuclein in lysates treated with α-synuclein antibodies for 48 h were 2.8 ng/ml with mAb49/G treatment (*p<0.04), 3.9 ng/ml with mAb211 (*p<0.05) and 3.3 ng/ml with mAb5C2 (*p<0.05), indicating reduced α-synuclein levels in cell lysates with antibody treatment (Fig. 5A).

In conditioned media from untreated cells, α-synuclein levels were calculated to be 6.9 ng/ml (Fig. 5B). In media from cells treated with α-synuclein antibodies for 48 h, α-synuclein levels were 3.2 ng/ml after treatment with mAb49/G (*p<0.05), 2.2 ng/ml with mAb211 (*p<0.05) and 2.3 ng/ml (*p<0.05) with mAb5C2 (Fig. 5B).

## Discussion

The use of immunotherapy to prevent or clear abnormal protein aggregates has emerged as a promising tool to treat neurodegenerative diseases. Also, disorders with aggregated α-synuclein may be targeted with immunotherapy and active immunization in transgenic mice has indeed been shown to reduce the accumulation of α-synuclein in the brain [22]. Although in that study it was proposed that immunization resulted in degradation of α-synuclein via the lysosomal pathway, it is still largely unknown by which mechanisms intraneuronal α-synuclein aggregates can be cleared [22].

There is an ongoing debate whether extracellularly administered antibodies can enter the cell and affect intracellular pathology. Indeed, antibodies utilized in cancer research have been shown to effectively bind to its target after cell internalization [26]. In addition, more recent work showed that antibodies directed against AβPP can maintain its structure and remain associated with its target after internalization [20]. In the present study we could detect α-synuclein antibodies within the cells after 48 h (Fig. 3D, E and F) of incubation and find that they co-occurred with the α-synuclein dimers/oligomers (Fig. 2D and 2E). Furthermore, our control experiments suggested that the presence of permeabilization reagents did not affect antibody internalization, thus indicating that the antibody uptake was not an effect of DNA transfection (data not shown).

Although our study indicates that α-synuclein can be targeted intracellularly, aggregated soluble species may also be possible to target in the extracellular space. Indeed, several recent studies on cells and transgenic mice have indicated cell-to-cell propagation of α-synuclein pathology [8], [16], [27]. In addition, neuropathological analyses of Parkinson's disease brains that had been transplanted with fetal mesencephalic dopaminergic neurons displayed Lewy bodies in the grafted cells, suggesting a similar propagation mechanism in the human brain [28].

Our findings demonstrate that extracellularly added α-synuclein antibodies reduced α-synuclein dimerization/oligomerization in living cells. Previously, in vitro studies have shown that α-synuclein aggregation can be decreased by expressing single chain fragments, i.e. intrabodies, targeting the C-terminus of α-synuclein [29].

We found that both the oligomer-selective antibody mAb49/G and the mAb211 antibody, raised against the C-terminal part of α-synuclein (epitope 121-125), were efficient in reducing α-synuclein dimerization/oligomerization (Fig. 4D and 4E). On the contrary, the mid-region antibody mAb5C2 did not significantly reduce the degree of dimer/oligomer formation. As some of the treated cells appeared to be somewhat smaller in size compared to untreated cells (-mAb) (Fig. 2 and 4), we also applied brightfield microscopy to better delineate the cell borders. By doing so, we found that there was no apparent difference in cell size between antibody treated and untreated cells (data not shown).

The inhibiting effect on oligomerization by mAb49/G was somewhat expected as we believe that this antibody recognizes an epitope exclusively present in the oligomeric structure of α-synuclein. Moreover, the efficient prevention with the C-terminal α-synuclein antibody was also not entirely surprising. We and others have described that oligomers and fibrils of α-synuclein expose C-terminal epitopes [11], [30] and α-synuclein antibodies directed against such epitopes are more efficient in clearing α-synuclein pathology in transgenic mice [23]. Along the same lines, the lack of effect on lowering dimer/oligomer levels for the 5C2 antibody in the current study could be explained by the fact that its hydrophobic NAC-region epitope (61–95) is hidden in the oligomeric core [31].

To further investigate antibody effects on α-synuclein oligomerization, we utilized ELISA to measure levels of α-synuclein in cell lysate and conditioned media from wells under the various experimental conditions. In previous studies, passive administration of Aβ and α-synuclein antibodies to cell and animal models have indicated antibody-directed protein clearance via the endosomal/lysosomal pathway [20], [23] In agreement with these findings, we showed that the levels of α-synuclein were decreased both in cell lysate and conditioned media after antibody treatment, pointing to an increased degradation of the targeted proteins (Fig. 5A and 5B). Possibly, the effects seen in our and the previous studies may be explained by antibody uptake via particular receptors. For example, the IgG-receptor tripartite motif-containing 21 (TRIM21) was shown to mediate antibody internalization followed by transferring of the antibody-antigen complex to the proteasome for degradation [32].

The finding that the NAC-specific 5C2 antibody influenced α-synuclein levels in both cell lysate and conditioned media without affecting oligomer formation is intriguing (Fig. 4F, 5A and 5B). Presumably, the 5C2 antibody fails to affect dimerization/oligomerization of α-synuclein but can bind to the monomeric α-synuclein hemi:GFP in which the NAC region is exposed. Thereby, also this antibody can facilitate protein turnover, thus explaining the decreased total α-synuclein levels seen in the ELISA. However, the oligomer-selective mAb49/G and C-terminal mAb211 antibodies would be more suitable antibody candidates, as they are targeting pathological α-synuclein aggregates rather than the physiological protein.

In summary, we have studied the effects of monoclonal α-synuclein antibodies on the early stages of oligomerization in H4 cells. We could show that extracellularly administered C-terminal and oligomer-selective α-synuclein antibodies are efficiently internalized, have an inhibiting effect on α-synuclein oligomer formation and facilitates protein turnover. Thus, these results provide further support for passive immunotherapy against α-synucleinopathies.
